# Anthropogenic landscape alteration promotes higher disease risk in wild New Zealand avian communities

**DOI:** 10.1371/journal.pone.0265568

**Published:** 2022-03-25

**Authors:** Antoine Filion, Lucas Deschamps, Chris N. Niebuhr, Robert Poulin

**Affiliations:** 1 Department of Zoology, University of Otago, Dunedin, New Zealand; 2 Department of Environmental Sciences, University of Quebec at Trois-Rivieres, Trois-Rivières (Québec), Canada; 3 Manaaki Whenua – Landcare Research, Lincoln, New Zealand; University of Oklahoma Norman Campus: The University of Oklahoma, UNITED STATES

## Abstract

Anthropogenic changes can have dramatic effects on wild populations. Moreover, by promoting the emergence of vector-borne diseases in many ecosystems, those changes can lead to local extinction of native wildlife. One of those diseases, avian malaria, has been shown to be on the rise in New Zealand, threatening native bird species that are among the most extinction-prone in the world. It is thus of prime importance to better understand the potential cascading effects that anthropogenic modifications have on those fragile species. Here, we aim to test how long-lasting modification to regional environmental filters can subsequently alter local biotic filters, in turn promoting the emergence of avian malaria in New Zealand avian communities. To this end, we used Bayesian structural equation modelling to unravel the drivers of disease emergence within the complex interplay between landscape and local species pools. We show that altered landscape, quantified through a lower enhanced vegetation index, leads to more infections in *Turdus spp*. and modification in avian community composition, potentially raising the probability of infection for other species in the community. In addition, we show that climatic variables associated with the presence of vectors play a predominant role in shaping the regional pattern of avian malaria occurrence. Our results suggest long-lasting impacts of anthropogenic changes on regional environmental filters and demonstrate that conservation efforts should align toward restoring the landscape to prevent further emergence of infectious diseases in wild ecosystems.

## Introduction

Emerging infectious diseases are a serious threat to native species that have not evolved with them [1]. In recent decades, the rise of those diseases has been increasingly linked to environmental anthropogenic change. Indeed, the distribution of infectious diseases is controlled by a complex interplay of environmental filters, such as climatic variables and landscape structure, controlling species distributions and assemblages at a local scale, the latter in turn acting as a biotic filter [2, 3]. By controlling the rate and spread of diseases, those filters act as a barrier to rapid disease emergence and evolution, ultimately shaping the disease landscape [2]. Vector-borne diseases, i.e. pathogens transmitted among vertebrates by a vector, are particularly prone to regulation by those filters [2, 4]. For example, the distribution of West-Nile virus, a disease of birds vectored by mosquitos, is constrained by a combination of abiotic factors at regional scales and avian assemblage composition at local scales [3], thus demonstrating that both environmental and biotic factors regulate emergence of vector-borne infectious diseases in the wild.

However, anthropogenic changes to various components of the environmental filter can increase the spread of those diseases. Global climate change, species translocations and changes in land use can all modify species composition at local scales [5]. With rapid shifts in community composition and changes in local habitat niche breadth, species that were once restricted to specific areas are now interacting with a wide range of other species, with pathogens spilling over among them. By changing the ecological function of the landscape, anthropogenic modifications have a direct impact on habitat availability and suitability for wildlife [6]. The resulting variation in environmental filters drive animal community composition at both local and regional scales [7–9]. At the local scale, habitat conversion may favour ubiquitous species and reduce species diversity [10, 11], with generalist host species more likely to occur across disturbed environmental matrices. In turn, by acting as established disease reservoirs, generalist species heighten the risk of constant spillover to native populations [2]. This phenomenon can have drastic impacts on individuals from populations that have not coevolved with diseases. With no co-evolutionary history in new host-pathogen associations, changes in disease virulence are likely in new hosts, potentially leading to local extinction of native wildlife [12, 13]. Additionally, at a community level, interspecific interactions between the introduced reservoir populations and susceptible native populations can boost the biomass of suitable disease hosts in an ecosystem, and therefore lower the threshold density needed for disease transmission within the system, which could lead to local extinction of native wildlife [14, 15]. Thus, vector-borne pathogens provide an ideal model to investigate the effects of anthropogenization of the landscape on disease emergence in wildlife.

Avian malaria is one of such vector-borne diseases, involving a haemosporidian pathogen (*Plasmodium* spp.) of birds vectored by biting dipterans, such as mosquitoes, that has been shown to be on the rise worldwide [16]. Indeed, avian malaria has received increased attention as it has been implicated as one of the main causes of extinction in native bird populations, mostly on islands [17]. For instance, Hawaiian birds provide the best example of the catastrophic effect malaria can have on already threatened bird populations. Following the accidental introduction of an ornithophilic mosquito vector (*Culex quinquefasciatus*) of the malarial parasite *Plasmodium relictum*, local hunter clubs imported many non-native bird species to the islands of Hawaii, probably without screening them for diseases. This in turn lead to a wave of native bird extinctions, even changing the spatial pattern of distribution of native Hawaiian species from low altitude to high altitude, a shift presumably allowing them to avoid the vector [18]. Even now, avian malaria is still playing a major role in native bird declines at their distributional limit, the latter being inversely correlated with mosquito abundance [17, 19].

New Zealand native bird species face a similar challenge; over the past centuries, as many as 137 non-native bird species were introduced to New Zealand, of which 28 species remain and now have well established populations [20], acting both as competitors for resources as well as spillover agents of avian malaria. Biotic interactions between populations are known to alter avian malaria patterns of transmission [21], and New Zealand avifauna is no exception. In fact, populations of introduced birds with wide niche breadth in New Zealand ecosystems, such as the blackbird (*Turdus merula)*, song thrush (*Turdus philomelos*) and house sparrow (*Passer domesticus*) have been shown to harbour high prevalence of avian malaria-causing parasites, potentially acting as reservoirs for the spillover of the disease toward native birds and increasing transmission rates in the wild [22, 23]. In addition, the historical accidental introduction of *Culex quinquefasciatus*, a highly adaptive and invasive mosquito and known vector for avian malaria, could also increase avian malaria transmission risk in the wild, especially in the warmer parts of the country [22, 24]. Moreover, recent studies have highlighted the potential role of avian malaria in the decline of native New Zealand bird species, which are already considered to be among the most extinction-prone in the world [25–27]. It is thus of prime importance to identify the underlying drivers of the spread of avian malaria in New Zealand.

**Fig 1:**
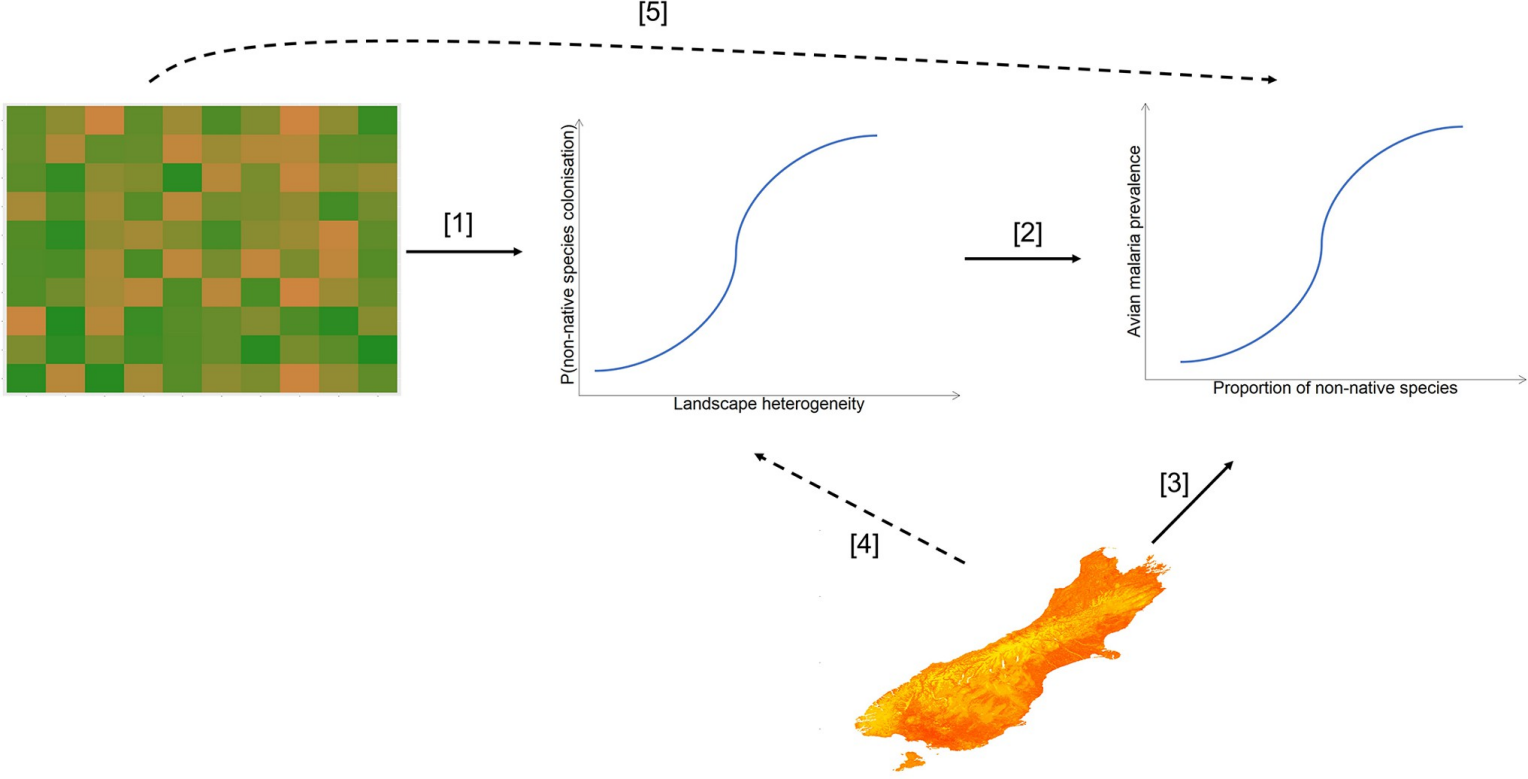
Theoretical framework of processes driving disease prevalence in New Zealand avifauna. Solid lines indicate effects predicted to be significant, whereas broken lines indicate non-significant effects. Higher landscape heterogeneity leads to an increased probability of colonization by non-native generalist birds [1], which leads to a higher prevalence of avian malaria [2]. Local climatic conditions determining the presence and activity of mosquitos will also influence the prevalence of avian malaria [3], but will have no impact on colonization by non-native birds [4]. Finally, landscape features are expected to have no direct influence on the prevalence of avian malaria [5].

In this light, structural equation modelling (SEM) can reveal both direct and indirect links between landscape structure and avian malaria transmission. As these pathogen transmission dynamics are known to be altered by both regional environmental and local biotic processes [16, 28], we take advantage of recent advances in remote sensing that now allow for multiple vegetation and landscape features to be readily available for most of the globe and combine these tools with sampling of local communities. Here, we aim to understand the role of both environmental and biotic filters in the spread of avian malaria in a temperate climate, the South Island of New Zealand. We predict that altered landscapes, represented by higher vegetation heterogeneity, lead to an increased probability of colonization by non-native generalist bird species (arrow 1 in the modelling framework presented in Fig 1). In turn, this community shift will result in a higher probability of avian malaria occurrence (arrow 2). Moreover, local environmental conditions such as temperature and rainfall that determine the emergence, presence and/or activity of mosquitos [29, 30], will contribute to shaping the disease landscape. We thus predict that bird populations from warmer and wetter parts of New Zealand will have a higher prevalence of avian malaria (arrow 3). We also predict that climatic drivers will have no impact on the colonization of non-native bird species (arrow 4), and that local avian communities will mediate the probability of avian malaria occurrence, which we expect to be completely independent from landscape features (arrow 5). We test those predictions with data from 18 sites along a landscape disturbance continuum on the South Island of New Zealand, from which we uncover cascading effects of landscape alteration on diseases in wild ecosystems.

## Methods

### Study system

The study system consists of conservation areas on the South Island of New Zealand (see online S1 Table for sampling locations). Over the past centuries, many anthropogenic changes, such as forest burning to provide grazing areas for sheep [31], have modified the landscape at local and regional scales, creating new ecological opportunities for invasive species to occupy but also supporting native species in the few patches of pristine forest left in those areas. With the establishment of multiple national parks and conservation areas, forested areas are now protected, and burning has long been abandoned. The result is a heterogenous landscape composed of few patches of pristine forest and large areas of tussock (i.e. grasslands) in some sites [31], with other sites remaining largely pristine (i.e. untouched forests), thus providing an ideal ecological framework to test our predictions.

### Study area and sampling

Sampling was conducted at 18 locations on the South Island of New Zealand during the austral summer (between late October 2020 and early January 2021), to ensure that most bird species would be in their breeding period; this maximised the chances of sampling individuals positive for avian malaria [32]. Each site was sampled only once. Bird sampling at each site took place on a single day, with three mist-nets set up at around 5:00 and taken down around 14:00. All birds were captured using passive mist-netting. All birds were identified to species level and numbered with metal bands to prevent re-sampling. After swabbing with 70% ethanol, we used a 27g needle to puncture the brachial vein of each captured individuals and collected ~ 0.1ml of blood, which was then placed unfrozen in a 1.5 ml Eppendorf tube containing 0.5 ml of Queen’s Lysis buffer [33]. All sampling was undertaken under New Zealand Department of Conservation wildlife authorisation act # 87614-FAU and University of Otago Animal Ethics permit # AUP-20-90.

### Parasite detection

We used a Qiagen DNeasy Blood and Tissue kit (QIAGEN, Valencia, California, USA) to extract DNA from unfrozen avian blood in buffer. We then screened samples for *Plasmodium* spp. presence using a nested PCR protocol to amplify a 478 bp sequence on the cytochrome *b* gene following the general guidelines of [34], with the notable exception of replacing TAQ polymerase with MyFi DNA polymerase to improve detection accuracy. The 1^st^ round of PCR used HaemNF1 and HaemNR3 primers and the nested PCR used HaemF and HaemR2 primers (only *Plasmodium* spp. have been detected in New Zealand; [35]). All samples were screened twice to reduce the risk of false negatives.

### Climate and vegetation data

To include environmental filtering in our analysis, we used the Enhanced Vegetation Index (EVI) as a continuous proxy of land use [36], but also because landscape structure is known to affect vector-borne diseases by regulating the presence or abundance of vectors and hosts [37, 38]. The EVI index ranges from -1, which corresponds to water/human structure, and 1 which corresponds to extremely productive areas. We accessed MYD13Q1 product Terra sensor of MODIS and requested 16 days averaged EVI databases at 500m resolution with a timespan encompassing the sampling period [39]. As none of our sites are near farmlands, which can substantially bias productivity indices, we believe that the use of this index is appropriate to measure vegetation differences between continuous pristine forest and previously disturbed area [36].

We extracted mean annual temperature and mean annual precipitation at a local scale (0.5° resolution) at each of the 18 locations from the WorldClim database [40].

### Biotic filter data

To evaluate the influence of host community composition on the occurrence of avian malaria in the wild, we recorded all passerine bird calls for a period of one hour (from 5:00 to 6:00) at each site on the day of sampling. In addition to the bird species identified by their call, we included any species that was seen during sampling or that was captured in mist-nets, and used the combined species list to build a presence/absence community matrix of 18 sites * 17 species that was then used as a predictor variable.

### Phylogenetic analysis

All analyses were performed in R version 4.0.0 [41]. To account for trait based evolutionary interplay between hosts and diseases [42], we built a phylogenetic tree using 1000 avian phylogenetic trees from BirdTree.org built with the backbone tree from [43]; we then used a random sample of 100 trees to account for phylogenetic uncertainty. We also tested whether avian malaria is more likely to occur in bird species from certain parts of the tree than in others, by quantifying the phylogenetic signal of avian malaria across bird species sampled, based on Pagel’s lambda (λ) using the *phylosig* function in the *phytools* package [44].

### Data analysis

We used Bayesian multi-level models (package: *Brms*; [45]) in a SEM framework to evaluate how environmental filters (landscape) affect local biotic filters (species community composition), in turn affecting the probability of local avian malaria occurrence (i.e.: we cannot rule out that sites with no infection found are truly free of infection).

### A side note on species community

Using the raw presence/absence host community matrix as predictor comes with its own challenges. For instance, including the whole matrix as predictor resulted in overfitted models, whereas using factor analysis (e.g. PCA or NMDS) axes dilutes inferences that can be derived from the presence of individual species. To overcome this problem, we ran 100 random subsets of the community matrix, preserving the 18 sites in all subsets but altering species composition by a maximum of five species per site, as it was the maximum number of species that could be included without further divergent transition/convergence issues. We then ran each of those communities as predictor with avian malaria occurrence per site as response variable (Bernoulli distribution). Afterward, we used PSIS-loo (pareto smoothed importance sampling leave-one-out; package *loo*; [46]), using the stacking method, to compare the predictive power of each individual model. Interestingly, all models selected using model averaging included a common species, *Turdus merula*. As it seemed to clearly be the most influential species of the whole community, we decided to only include the presence/absence of this species as a predictor in our final models.

### Final models

We used a Bernoulli distribution for both our response variables in the SEM. We used informative priors adjusted on the sample mean for intercept and population-level effect. Each SEM model was first built with a direct causal link from landscape (EVI, scaled value) toward species community (*Turdus merula* presence probability) and then toward the probability of avian malaria occurrence. We then defined 3 more complex SEM models that could be defined *a priori* based on our knowledge of the system and data availability. The first only incorporated an indirect link from EVI to avian malaria occurrence, whereas the second and third one incorporated climatic data (either mean annual precipitation or mean annual temperature, scaled values) at 0.5° resolution. Direct links from climatic variables toward landscape index and species community were evaluated in separate multivariate models, as it was impossible to conserve those links without overfitting more complex SEM models. We ran all models using four chains of 15000 iterations each and set the *adapt_delta* function to 0.99999 and the *maximum_treedepth* to 15 to eliminate divergent transitions after warmup. For each model, we confirmed the convergence for each variable by checking the potential scale reduction factor on split chains (Rhat) indicator (at convergence, Rhat is equal to one). We then used leave-one-out cross validation with both stacking and pseudo-BMA method to select the best possible SEM model explaining the probability of avian malaria occurrence. If both credible intervals did not overlap with 0, we considered an effect to be relevant in the model.

## Results

**Fig 2:**
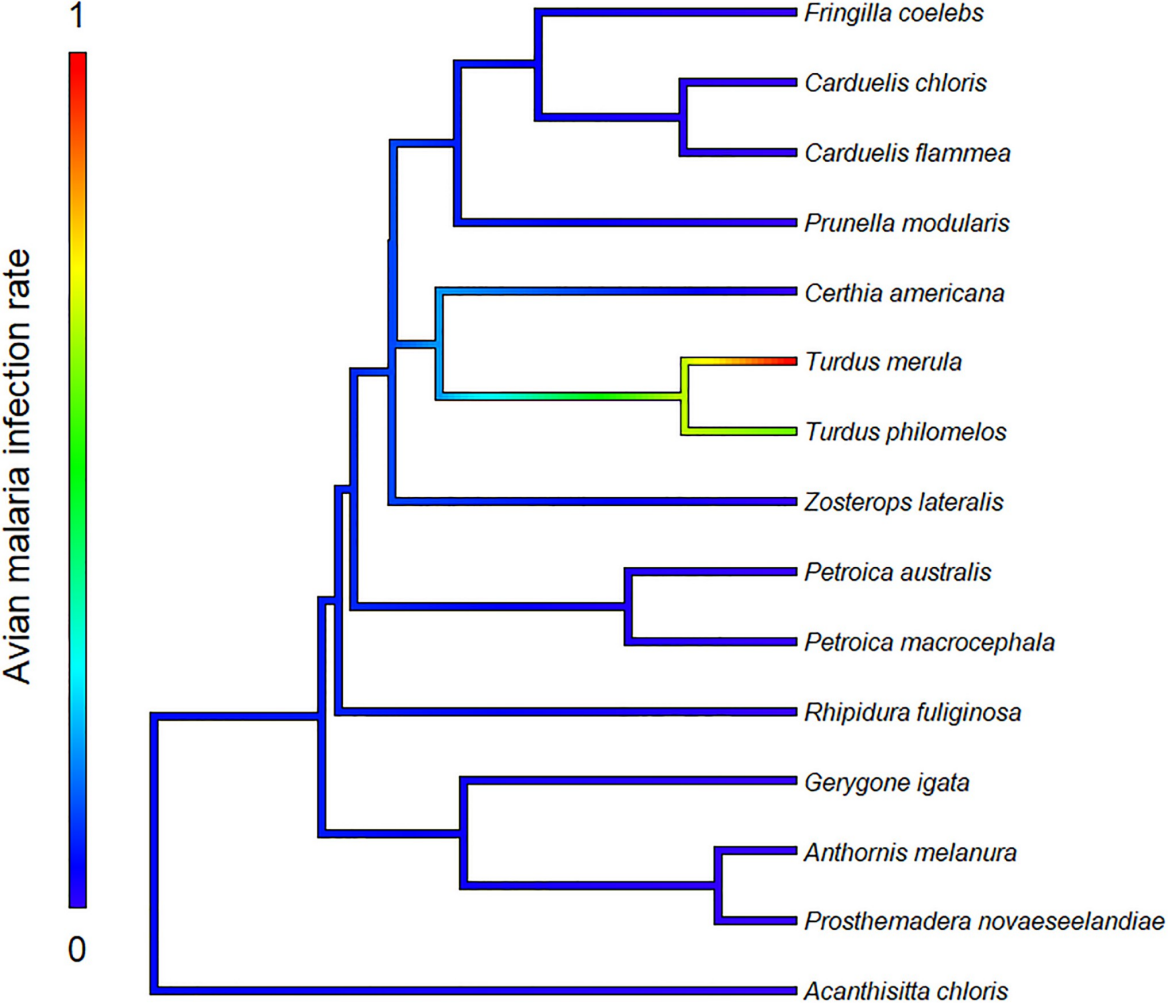
Phylogenetic distribution of avian malaria infections among all avian host species sampled during this study. Pagel’s lambda value of this phylogram is 0.985.

Overall, we collected 188 blood samples from nine native and six non-native passerine species (see online S1 Table for number of individuals of each species sampled per site). After PCR screening, we found seven *Plasmodium* spp. infections, five in *Turdus merula* and two in *Turdus philomelos*, thus unsurprisingly malaria infections exhibited a strong phylogenetic signal (λ = 0.985; Fig 2).

The three tested SEM models had relatively close expected log pointwise predictive density (ELPD) scores, all being in a range of four from each other. However, further model selection using leave-one-out cross validation identified that models including either local temperature (staking weight = 0.460, pseudo-BMA weight = 0.451) or precipitation (staking weight = 0.540, pseudo-BMA weight = 0.481) provided the best explanation for the variation in the probability of local avian malaria occurrence compared to the simpler one, containing only EVI (staking weight = 0.0, pseudo-BMA weight = 0.068). Since the 2 models incorporating climatic variables represent alternative explanations of avian malaria occurrence, we choose to present them as plausible alternatives.

**Fig 3:**
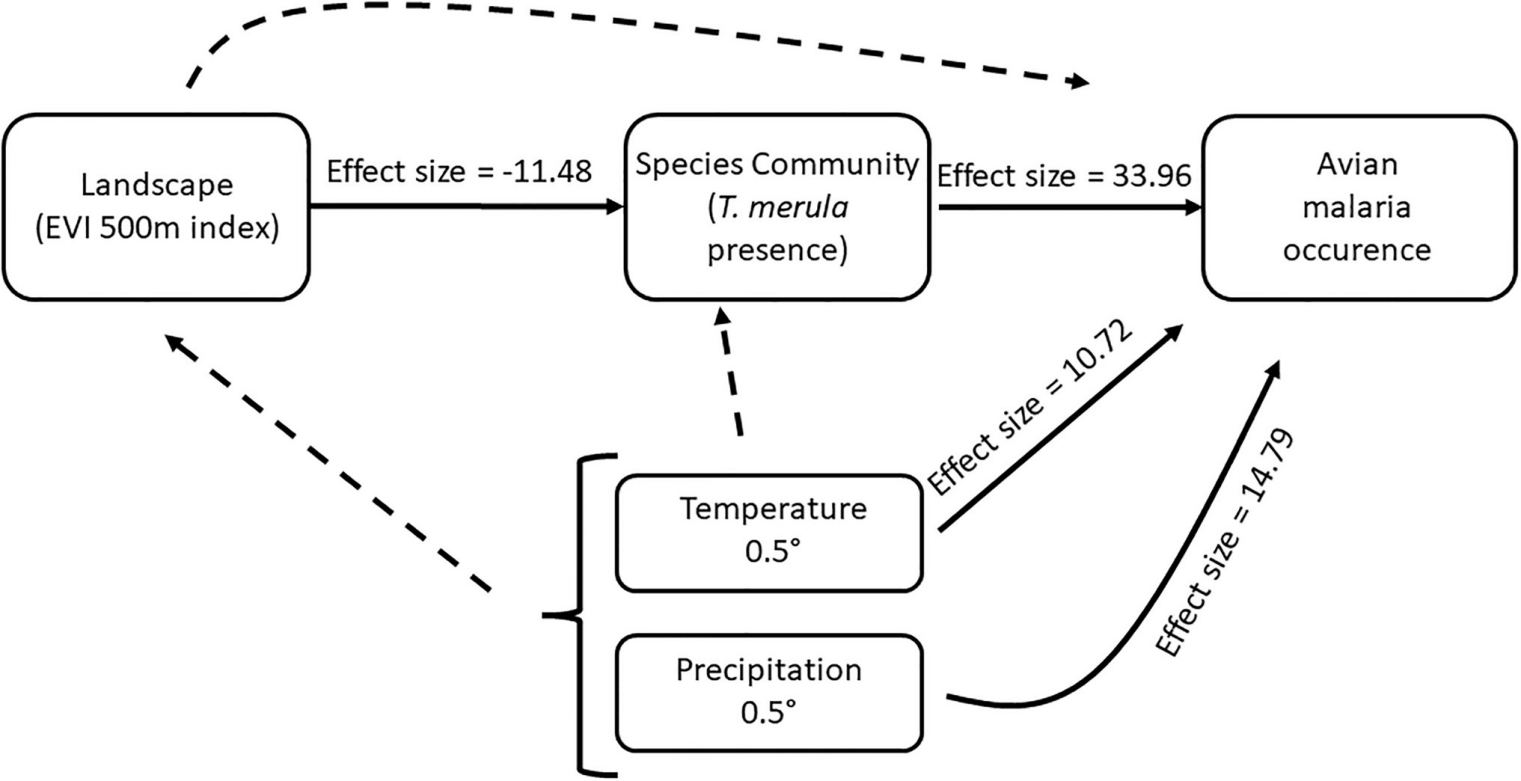
SEM diagram depicting all the tested relationships in the South Island of New Zealand ecosystem. We define an effect as relevant in the model if the 95% credible interval does not contain 0. Dashed lines represent relationships with no relevant effect and solid lines represent those with relevant effects.

The SEM analysis uncovered multiple effects among predictors in the network (see Fig 3). All effect sizes that were common across the three SEM models were extracted from the precipitation model, as it had the best fit among the three. Firstly, we found that EVI had a negative effect on the presence on *Turdus merula* (Effect size = -11.48, lower CI = -48.88, upper CI = -1.74), with this species more likely to be found in disturbed areas. In turn, the presence of that species had a positive effect on avian malaria occurrence probability (Effect size = 33.96, lower CI = 4.17, upper CI = 183.59). In addition, we found that both local mean annual temperature and precipitation had a positive effect on the probability of avian malaria occurrence (Temperature: Effect size = 10.72, lower CI = 1.13, upper CI = 47.55, Precipitation: Effect size = 14.79, lower CI = 1.30, upper CI = 81.63). As predicted, we failed to find any relevant residual effect of EVI on avian malaria occurrence (Effect size = -3.75, lower CI = -23.73, upper CI = 5.26) nor any direct effect of climatic variables on either EVI (Temperature: Effect size = -0.02, lower CI = -0.28, upper CI = 0.24, Precipitation: Effect size = -0.11, lower CI = -0.37, upper CI = 0.14) or *Turdus merula* presence (Temperature: Effect size = 2.42, lower CI = -2.19, upper CI = 13.37, Precipitation: Effect size = 1.15, lower CI = -4.41, upper CI = 9.48).

## Discussion

Our study shows that alteration in the environmental filter (landscape features) leads to increase in infection of *Turdus spp*. and alteration in the avian species composition, potentially influencing the probability of local avian malaria infection for other species in the community. In addition, we show that climatic variables known to affect the presence and activity of vectors of avian malaria also play a predominant role in shaping the disease transmission structure in New Zealand, with warmer and wetter localities having an enhanced transmission risk of avian malaria.

While the low overall prevalence of infection was unexpected, as many more species are known to be infected by avian malaria (*Plasmodium* spp.) in New Zealand (see review by [26]), it is not entirely surprising, as true generalist species, such as *Turdus* species [47], are known to be active reservoirs of vector-borne diseases [2]. The strong phylogenetic signal observed is also in contradiction with what has been observed in other systems, as larger scale studies have shown that *Plasmodium* spp. usually have a phylogenetic signal ranging from λ = 0.24 to λ = 0.35 [16, 21]. This could be due to the restricted spatial range of our study, encompassing relatively low host diversity, but it could also suggest an increased virulence of the disease in native species at regional scale, causing infected individuals to die or be captured at low rates. In fact, higher virulence of the disease, leading to increased mortality rates in new hosts, has been shown in many other vector-borne disease systems [13]. Increased mortality rates in native New Zealand species, due to enhanced virulence of avian malaria in those species could thus provide a possible explanation for both the low number of infected individuals sampled and the strong phylogenetic signal observed.

Our results suggest that landscape structure drives species community composition at a regional scale, resulting in a higher probability of *Turdus* spp. presence, potentially influencing local avian malaria occurrence, in accordance with our prediction. We acknowledge that one of our predicted and observed effects suffers from circularity, i.e. the presence of *Turdus* individuals influences the probability of finding local malaria infections, since only *Turdus* individuals were found to be infected in our samples. Since many other introduced and native birds in New Zealand are known hosts of malaria (see [26]), we did not expect this limited infection range. Nevertheless, two other observed effects provide insights into avian malaria dynamics: (i) landscape features, i.e. the EVI index, predict the spatial occurrence of *Turdus* spp., and (ii) climatic variables determine the probability that these reservoirs of malaria are locally infected. While we only included that single bird taxon from our community matrix in larger models, it nevertheless shows that niche availability, dictated by landscape features, directly influences species distribution and subsequent disease risk. Landscape features are known to be a major driver of community composition at regional scales [48]. Indeed, newly created niches in the habitat provide ecological opportunities for new species to colonize those environments [49]. By opening previously pristine habitats and restricted ecological niches, anthropogenic change to the landscape can create spillover of pathogens toward native populations [50], in this case mediated by the intrusion of *Turdus* reservoirs. For instance, [3] demonstrated that species composition of host assemblages was one of the key drivers of disease transmission success at a local scale for two emerging vector-borne diseases, West-Nile virus and Lyme disease. We believe that this phenomenon provides a suitable explanation in the case of avian malaria in New Zealand avifauna, as introduced species (such as *Turdus* species) exploit a different niche than native species [51]. Thus, broadening the ecological niche (i.e. higher fragmentation) has probably led to increased interactions and changes in local species pools, enhancing the chance of spillover. Our results also suggest that there is no underlying effect of the landscape on disease transmission once the local pool of species is considered, suggesting that it is not the landscape *per se* that influences avian malaria, but rather a more complex cascading interaction. Therefore, we believe that our approach allowed us to potentially identify one of the main biotic filters of avian malaria in New Zealand.

A central finding of this study is that both climatic variables used as proxies for the presence of vectors, namely mean annual temperature and mean annual precipitation, have an influence on avian malaria occurrence in the wild. This is in accordance with our prediction, as we expected warmer and wetter parts of the South Island of New Zealand to be more heavily infected due to the increased transmission probability between hosts. This result is also widely supported by the literature, as many other vector-borne diseases using mosquitoes as transmission vectors follow the same pattern [52, 53]. For example, cases of St. Louis encephalitis virus, another mosquito-borne disease, have been shown to be more prevalent during wetter years in the southern United States [54]. The fact that we found that local mean annual temperature influences the probability of avian malaria occurrence could be due to the pathogen maturation time in the vector. From a vector’s perspective, local temperature could play a key role in avian malaria development time in the vector, as maturation times are shorter in warmer conditions [55], thus enabling increased local transmission potential. Moreover, the increase in avian malaria prevalence in wetter and warmer parts of New Zealand could be related to mosquito density, as local climatic conditions are known factors contributing to an increase in mosquito development rates [29, 56], providing a suitable explanation to the pattern we observed. Put in a broader conservation context, as warming events resulting from climate change continue to affect many ecosystems, this could result in an increase in avian malaria into an already disturbed and fragile avifauna, which could potentially lead to local extinction of threatened species.

In conclusion, we show that anthropogenic changes can have dramatic cascading effects on diseases in a wild ecosystem. Of course, there are many limitations to this study which should be considered when interpreting these results. For instance, the limited number of predictors that could be included in the model does not allow complex interactions to be identified; these could arise when considering whole species assemblages instead of only one key species as a proxy of community composition. In addition, we resorted to proxies instead of using the actual variables that may influence avian malaria occurrence (i.e., EVI for landscape features and climatic variables instead of actual mosquito abundance), which limits the inferences that can be made from our results. Moreover, our small number of avian malaria positive individuals, restricted to two species of the same genus, limits our power to make accurate predictions of avian malaria infections across the avian community. Nevertheless, even with our relatively small dataset, we were able to demonstrate clear patterns of avian malaria occurrence in wild New Zealand avifauna. More specifically, we provide evidence of long-lasting effects of anthropogenic changes on the emerging infectious diseases landscape. In the wake of global conservation efforts, we posit that landscape restoration is one of the key factors mediating the impact of emerging infectious diseases in wild ecosystems.

## Supporting information

S1 Table Summary of all passerine species captured in mist-nets at each sampled site in the South Island of New Zealand. (DOCX)

S1 Data (CSV)
