# Supplementary material for: Anthropogenic landscape alteration promotes higher disease risk in wild New Zealand avian communities
Source: PLoS One. 2022 Mar 25;17(3):e0265568. doi: 10.1371/journal.pone.0265568 (PMC8956180; doi:10.1371/journal.pone.0265568)

**S1 Table. Summary of all species captured in mist-nets at each site in the South Island of New Zealand.**


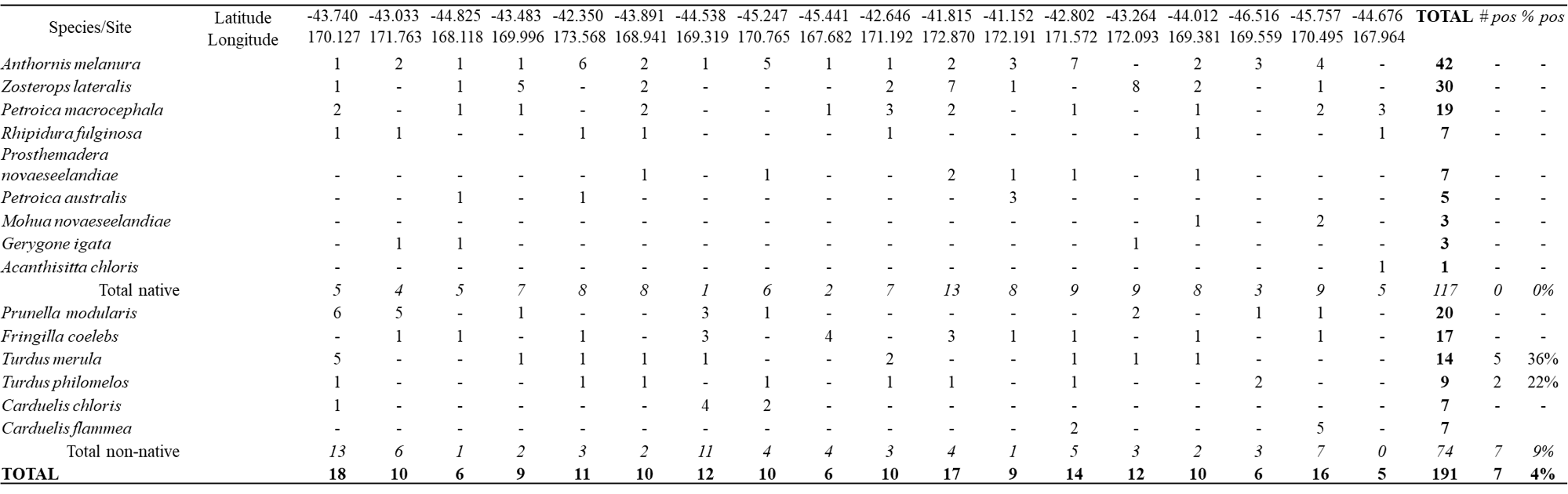

Supplement: S1 Table — (DOCX) [file pone.0265568.s001.docx]
